# Carotid artery stenting has similar outcomes in men and women

**DOI:** 10.1590/1677-5449.200169

**Published:** 2021-05-31

**Authors:** Marina Ansuategui, Gabriela Ibarra, Carmen Romero, Alejandra Comanges, Jose A. Gonzalez-Fajardo

**Affiliations:** 1 Hospital Universitario 12 de Octubre, Madrid, Spain.

**Keywords:** carotid artery stenting-female, complications, mortality, restenosis, stent de carótida-feminino, complicações, mortalidade, reestenose

## Abstract

**Background:**

The aim of carotid interventions is to prevent cerebrovascular events. Endovascular treatment (carotid-artery-stenting/CAS) has become established as an alternative to open surgery in some cases. Historically, female sex has been considered as a perioperative risk factor, however, there are few studies regarding this hypothesis when it comes to CAS.

**Objectives:**

To analyze the CAS results in our center adjusted by sex.

**Methods:**

A retrospective cohort study was designed, including patients with carotid atheromatosis operated at a single center from January 2016 to June 2019. Our objective was to compare cardiovascular risk, including myocardial infarction, stroke, and mortality, by sex. Follow-up rates of stent patency, restenosis, stroke, myocardial infarction, and death were reported.

**Results:**

71 interventions were performed in 50 men (70.42%) and 21 women (29.57%). Mean age was 70.50 ± 10.72 years for men and 73.62 ± 11.78 years for women. Cardiovascular risk factors did not differ significantly between sexes. Mean follow-up was 11.28 ± 11.28 months. There were no significant differences in neurological events during follow-up. No adverse cardiological events were detected at any time. Regarding the mortality rate, during medium-term follow up there were 2 neurological related deaths with no significant differences between sexes (p=0.8432). Neither sex had higher rated of restenosis during long term follow-up (5.63% *vs.* 1.41%, p = 0.9693) or reoperation (1.41% *vs.* 1.41%, p = 0.4971). All procedures remained patent (<50% restenosis).

**Conclusions:**

Despite the limitations of our study, CAS is a therapeutic option that is as effective and safe in women as in men. No sex differences were observed.

## Resumo

**Contexto:**

As intervenções carotídeas visam prevenir eventos cerebrovasculares. O tratamento endovascular (implante de stent na artéria carótida) se estabeleceu como uma alternativa à cirurgia aberta em alguns casos. Historicamente, o sexo feminino é considerado um fator de risco perioperatório, mas há poucos estudos abordando essa hipótese em casos de stent de carótida.

**Objetivos:**

Analisar os resultados do CAS em nosso centro ajustados por sexo.

**Métodos:**

Este estudo de coorte retrospectivo incluiu pacientes com ateromatose carotídea operados em um centro de janeiro de 2016 a junho de 2019. Nosso objetivo foi comparar o risco cardiovascular, incluindo infarto do miocárdio, acidente vascular cerebral e mortalidade, de acordo com o sexo. No seguimento, foram descritos patência do stent, reestenose, acidente vascular cerebral, infarto do miocárdio e morte.

**Resultados:**

Setenta e uma intervenções foram realizadas: 50 homens (70,42%) e 21 mulheres (29,57%). A média de idade foi de 70,50 ± 10,72 anos nos homens e 73,62 ± 11,78 anos nas mulheres. Os fatores de risco cardiovascular não diferiram significativamente entre os sexos. A média de seguimento foi de 11,28 ± 11,28 meses. Durante o seguimento, não houve diferenças significativas nos eventos neurológicos. Nenhum evento cardiológico adverso foi detectado. Quanto à taxa de mortalidade, durante o seguimento de médio prazo ocorreram 2 óbitos neurológicos sem diferenças significativas entre os sexos (p = 0,8432). Não foi observada maior taxa de reestenose no seguimento de longo prazo (5,63% vs. 1,41%, p = 0,9693) ou de reoperação (1,41% vs. 1,41%, p = 0,4971) ao comparar os dois sexos. Todos os procedimentos permaneceram pérvios (< 50% de reestenose).

**Conclusões:**

Apesar das limitações deste estudo, o stent de carótida em mulheres é uma opção terapêutica tão eficaz e segura quanto em homens. Nenhuma diferença foi observada entre os sexos.

## BACKGROUND

The aim of carotid procedures is to prevent cerebrovascular events. Currently, carotid artery stenting (CAS) seems to be a valid choice in certain patients (severe heart failure, severe pulmonary disease, previous radiation therapy to the neck, recurrent stenosis after endarterectomy) compared to open surgery.^1^^,^^2^

The beneficial results of open surgery (CEA: Carotid Endarterectomy Artery) in women have been questioned, leading to some uncertainty about the effect of female sex in endovascular therapy.

Several studies have associated female sex with adverse outcomes, such as higher rates of perioperative stroke,^3^ restenosis, or reintervention.^4^ Some of the features that could negatively influence prognosis in this group are vessel size, plaque morphology, sensitivity to antiplatelet agents, and sex hormones.^5^ Nevertheless, other authors have not shown a relationship between these adverse events and female sex.^6^^,^^7^

## METHODS

Considering the uncertainty and contradictory results on this topic, the primary objective of this study was to compare cardiovascular risk (heart attack or stroke) and mortality after CAS according to sex. Secondary endpoints analyzed patency, restenosis, and reintervention.

A retrospective cohort study was designed, including consecutive patients with carotid atheromatosis operated at our center from January 2016 to June 2019.

Accepting an alpha risk of 0.05 and a power of 80% to detect a medium effect size (w = 0.35), at least 68 subjects were necessary.

All patients underwent preprocedural cerebrovascular imaging (CT-angio or MRI-angio). All symptomatic patients had been evaluated by a neurologist and patients with both acute and established strokes were included. Patients with significant asymptomatic carotid stenosis (≥70% graded by duplex ultrasound) in whom CEA was contraindicated (comorbidities: ≥85 years old, severe heart failure, hostile radiated neck, and restenosis) were also included. Patients who underwent CAS due to dissection, trauma, or fibromuscular dysplasia and patients who did not adhere to postoperative medical therapy (double antiaggregation and statins) were excluded. The study was approved by the Hospital’s Research Ethics Committee.

Demographic data, neurological status, and comorbidities were recorded. Rates of patency, restenosis, stroke, angina-myocardial infarction, and death during the perioperative (30 days) and postoperative period were examined according to sex (female *vs.* male) and confirmed by the pertinent imaging exams and laboratory tests.

Individual patient data were obtained through the hospital’s electronic medical records. Due to the retrospective nature of the study, previous diseases were defined according to the criteria and treatment reported by the patient on admission.

Risk factors reviewed were: age, sex, smoking history, diabetes, hypertension, dyslipidemia, atrial fibrillation, chronic obstructive pulmonary disease, chronic kidney disease, and stroke. Acute myocardial infarction and angina (stable or unstable) were grouped into the variable ischemic heart disease. Furthermore, whether patients had undergone carotid surgery or cervical radiotherapy and if they had received anticoagulant or antiplatelet therapy prior to the intervention was also recorded. All patients received statins according to established hospital protocols. Additionally, analysis was also conducted on the basis of whether the intervention was elective or urgent (stroke unit).

Patients were considered symptomatic if they had been evaluated by neurology, developed cerebral ischemic symptoms during the 6 months prior to the procedure (amaurosis fugax, other transient ischemic attacks, or established ischemic stroke),^8^ or had objective brain imaging evidence of ischemia-infarction (CT-angio or MRI-angio).

The degree of carotid stenosis was determined according to the University of Washington hemodynamic criteria,^9^ validated by our hospital, and following the recommendations of the Vascular Diagnosis Chapter of the Spanish Society of Angiology and Vascular Surgery (SEACV) guidelines.^10^

According to NASCET criteria^11^ and European Guidelines,^12^ selected symptomatic patients with stenosis ≥50% or asymptomatic patients with stenosis ≥70% were candidates for intervention.

An indication for CAS was established in patients with hostile neck (radiotherapy or previous surgery) or severe comorbidities that precluded them from open surgery (CEA), including ≥85 years old (taking into account life expectancy and preoperative functional status) or severe heart failure. CAS was also used in those patients managed in the stroke unit (acute clinical cases) who had significant carotid bifurcation stenosis at the time of intervention.

All CAS procedures were performed with double antiaggregation (acetylsalicylic acid-100mg and clopidogrel-75mg), or loading dose when applicable (clopidogrel-300mg), and a distal protection filter system (Spider FX®, FilterWire EZ®), using open cell, closed cell, or double-mesh stents according to the surgeon’s discretion (usually closed-cell or double-mesh stent for greater plaque coverage, although open-cell stents were used in strongly angled carotid bulbs for anatomic preservation).

During the immediate postoperative period (3 months),^13^ all patients received double antiplatelet therapy, except for anticoagulated patients, who received simple antiaggregation. Treatment with statins (atorvastatin 40mg) was indicated indefinitely.

Reintervention and death were reported according to clinical follow-up. The patency of the procedure was always evaluated by Doppler ultrasound at discharge, 1 month, 6 months, and 1 year after the procedure. Restenosis was considered present if the patient had stenosis ≥50% of the treated artery, measured by Doppler ultrasound, according to NASCET criteria^11^ (CCA/ICA ratio between 2 and 4), in any follow-up period.

Quantitative variables were expressed as mean ± standard deviation and interquartile range; these were compared using *t* tests. Qualitative variables were expressed as absolute and relative frequencies and were compared using chi square tests. Kaplan-Meier analyses were used to estimate survival function, stroke, or postoperative myocardial infarction, as well as restenosis and reoperation rate between the sexes. Male/female curves were compared using Log-rank tests. All analyses were performed using SAS statistical software, version 9.4 of the SAS System for Windows. Copyright © 2002-2012 SAS Institute Inc.

## RESULTS

Between January 2016 and June 2019, 71 interventions were performed in high risk patients at our center in collaboration with Interventional Neuroradiology. Fifty (70.42%) of these patients were men and 21 (29.57%) were women. Mean age was 70.50 ± 10.72 years (89-51) for men and 73.62 ± 11.78 years (89-53) for women (p = 0.31).

All the interventions were accomplished via percutaneous femoral access.

Cardiovascular risk factors did not differ significantly between men and women (Table 1). Rates of diabetes, dyslipidemia, and hypertension were similar in both groups. Almost 50% of men and women had suffered a previous cerebrovascular event. The rate of previous myocardial infarction was higher in men (34% *vs.* 19%) and the rate of atrial fibrillation was higher in women (8% *vs.* 14.3%). Clinical presentation was comparable between both groups (Table 1): 40% of men had acute symptoms *vs.* 42.9% in women.

**Table 1:** Patient characteristics and stent type.

	**Carotid Artery Stenting**			p*
	Total (n =71)	Men (n=50)	Women (n=21)	
	N (%)	N (%)	N (%)	
Age				
Mean ± SD		70.58 ± 10.72 (89-51)	73.62 ±11.78 (89-53)	0.3100
Smoking status (N=62)				
Current	18 (29.03)	13 (28.26)	5 (31.25)	0.7541
Former	28 (45.16)	22 (47.83)	6 (37.5)	
Hypertension	53 (74.65)	36 (72.0)	17 (80.95)	0.4287
Diabetes	27 (38.03)	18 (36.0)	9 (42.86)	0.5870
Dyslipidemia (N=70)	46 (65.71)	32 (65.31)	14 (66.67)	0.9125
CKD	13 (18.31)	8 (16.0)	5 (23.81)	0.4374
COPD	9 (12.68)	8 (16.0)	1 (4.76)	0.1940
Previous stroke (6 months)	36 (50.70)	25 (50.0)	11 (52.38)	0.8547
CAD	21 (29.58)	17 (34.0)	4 (19.05)	0.2077
Atrial fibrillation	7 (9.86)	4 (8)	3 (14.29)	0.4175
Anticoagulant therapy	9 (12.68)	6 (12)	3 (14.29)	0.7916
Urgent	29 (40.85)	20 (40)	9 (42.86)	0.7979
Previous CEA	10 (14.08)	7 (14.0)	3 (14.29)	0.9748
Previous RT	14 (19.72)	12 (24.0)	2 (9.52)	0.1618
Severe comorbidities (≥85 years old, severe heart failure)	11 (15.49)	6 (12.0)	5 (23.81)	0.1268
Stent type				
Wallstent®	29 (40.85)	21 (42.0)	8 (38.1)	0.5451
Acculink®	7 (9.86)	6 (12.0)	1 (4.76)	
Roadsaver®	35 (49.30)	23 (46.0)	12 (57.14)	

SD: standard deviation, CKD: chronic kidney disease, COPD: chronic obstructive pulmonary disease, CAD: coronary artery disease, CEA: carotid endarterectomy, RT: radiotherapy.

* p value for age calculated by *t* test. For all other variables, p values calculated with chi-square tests.

Closed cell stents (Wallstent®) were used in 29 patients (40.85%), open cell stents (Acculink®) in 7 patients (9.86%), and double-mesh stents (Roadsaver®) were used in 35 patients (49.30%) (Table 1).

Thirty-six of the cases (50.7%) were symptomatic patients (p = 0.8547), although we also treated asymptomatic patients with hemodynamically significant carotid restenosis >70% (n = 10, 14.08%, p = 0.627), patients with previous radiotherapy (n = 14, 19.72%, p = 0.1618), and patients with severe comorbidities such as ≥85 years old or severe heart failure (n = 11, 15.5%, p = 0.1268).

Twenty-nine patients (20 men *vs.* 9 women) were urgently operated during a stroke code for an urgent ischemic cerebrovascular process, homogenously distributed between the two groups (p = 0.7979). In these cases, concomitant cerebrovascular thrombolysis/aspiration was performed when necessary.

Median follow-up was 11.28 ± 11.28 months.

First, we analyzed the 29 patients who underwent CAS during a stroke code (25 [86.20%] men *vs.* 4 [13.79%] women). Four patients in this subgroup died, all of them during the perioperative period (in these cases, during the first 3 days after treatment), with no significant differences in survival function between sexes (p=0.1556). There were no postoperative strokes during long-term follow up and no restenosis or reinterventions.

Six of the 42 patients (30 [71.4%] men *vs.* 12 [28.57%] women) who underwent scheduled CAS died (4 men *vs.* 2 women), as explained further below, without significant differences in survival function when neurological mortality was compared (p=0.8432).

**Figure 1:**
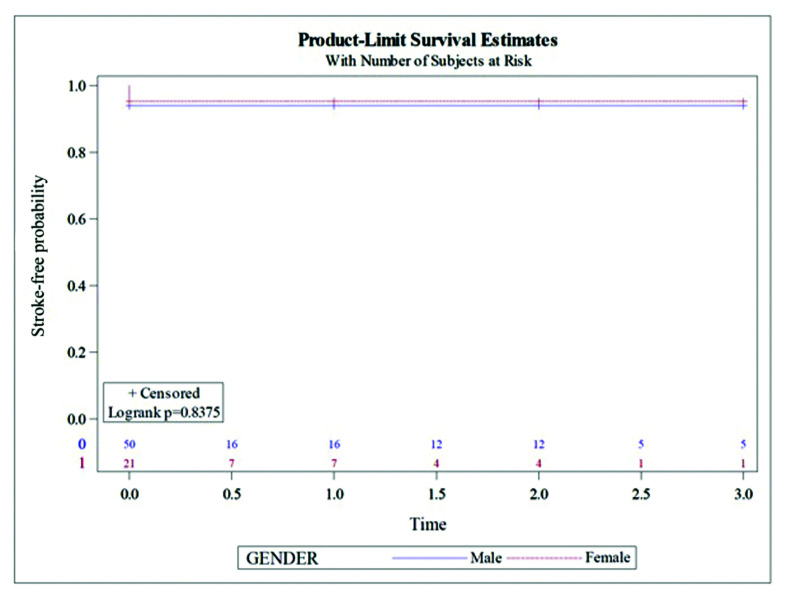
Neurological events during follow-up (time in years).

When all 71 patients were analyzed together, there were four postoperative strokes during short-term follow-up (3 men, 6% *vs.* 1 woman 4.76%) (Table 2), with no significant differences between sexes (p = 0.8375) (Figure 1). Two of these cases (1 man, 1 woman) were patients with a previous carotid surgery who underwent CAS because of restenosis and suffered a transitory ischemic attack (TIA) during the first month after the procedure. One patient suffered a TIA a few hours after stent implantation and the imaging exams didn’t reveal any defects regarding the stent. The fourth presented left hemiparesis related to a small stent thrombus that didn’t require treatment and was resolved during follow up with anticoagulation.

**Table 2:** Procedural outcomes (number of events during follow-up).

		**Total (n =71)**	**Men (n=50)**	**Women (n=21)**	p*
		N (%)	N (%)	N (%)	
Stroke		4 (5.63)	3 (6.0)	1 (4.76)	0.8375
MI		0 (0)	0 (0)	0 (0)	-
Restenosis		5 (7.04)	4 (8.0)	1 (4.76)	0.9693
Reintervention		2 (2.82)	1 (2.0)	1 (4.76)	0.4971
Total mortality		10 (14.08)	8 (16.0)	2 (9.52)	0.4681
Neurological mortality	Asymptomatic	0 (0)	0 (0)	0 (0)	-
	Symptomatic	6 (8.45)	4 (8.0)	2 (9.52)	0.9677
	Elective	2 (2.82)	1 (2.0)	1 (4.76)	0.8432
	Urgent	4 (5.63)	4 (8.0)	0 (0)	0.1556

MI: myocardial infarction.

* p values calculated with Log-rank tests.

No adverse cardiological events (0%) were recorded during the postoperative period or follow-up.

**Figure 2:**
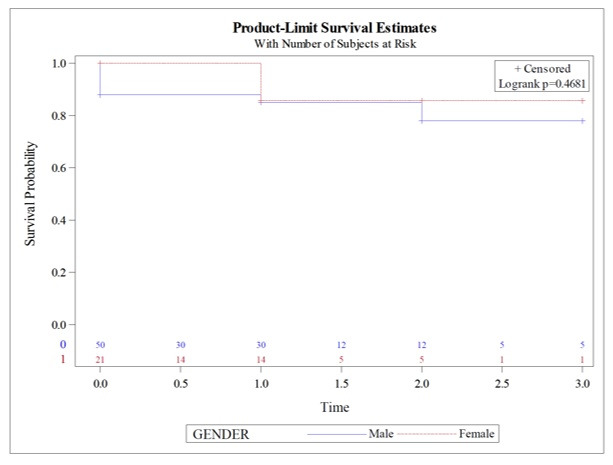
Overall mortality (time in years).

Regarding the overall mortality rate (Figure 2), ten patients (14.08%) died, with no significant differences in survival function between men and women (p=0.4681). Four patients died during the perioperative period (5.6%) (4 men *vs.* 0 women), all of whom were patients admitted on a stroke code who never awoke after carotid intervention. Six (8.5%) deaths (4 men *vs.* 2 women) were observed during the postoperative period (after 30 days) (Table 2). One was due to a known oncological illness, two due to cerebrovascular causes (hemorrhagic stroke), and three due to unrelated causes (respiratory and digestive).

**Figure 3:**
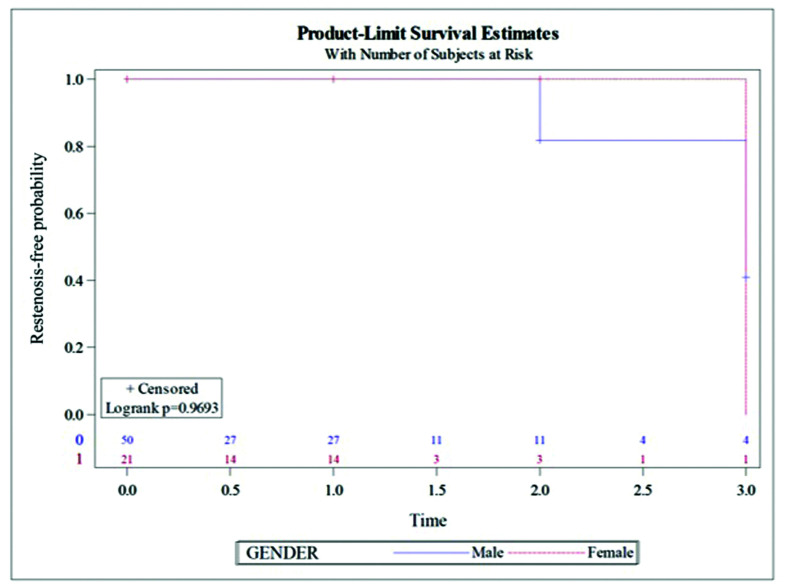
Restenosis rate (time in years).

**Figure 4:**
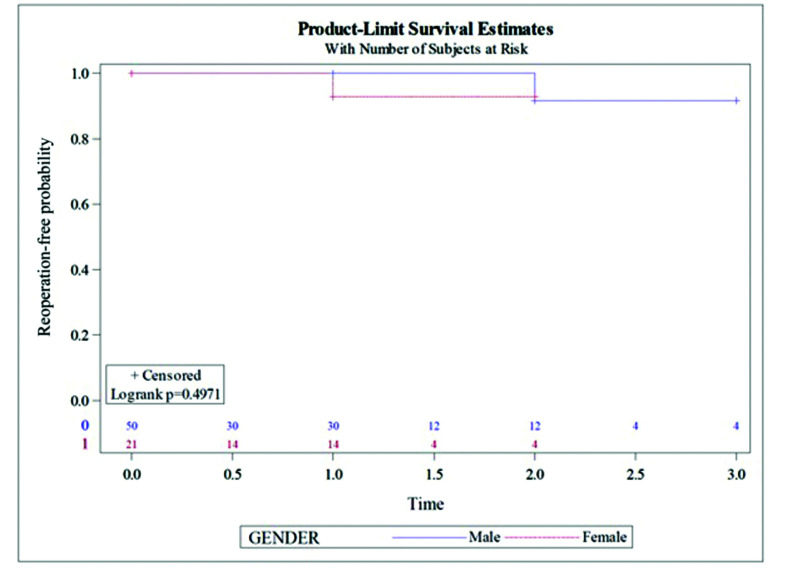
Reoperation rate (time in years).

During follow-up, neither sex exhibited a higher restenosis rate (8% *vs.* 4.76%, p = 0.9693) (Figure 3) or reoperation rate (2% *vs.* 4.76%, p = 0.4971) (Figure 4). These restenosis were detected during long-term follow-up. All procedures remained patent (<50% restenosis).

The total survival of the series was 85.9% and the proportion free from stroke-myocardial infarction was 94.37%, with no significant differences found.

**Figure 5:**
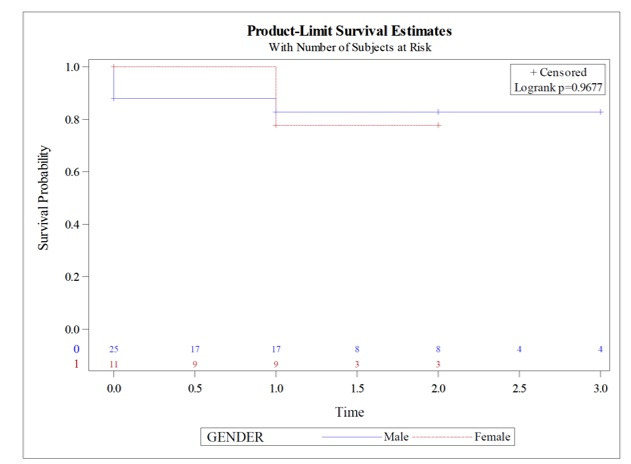
Mortality in patients with previous neurological symptoms (time in years).

When the subgroup of patients with previous neurological symptoms were analyzed, there were no significant differences between the sexes (mortality: six patients, 4 men *vs.* 2 women, p = 0.9677) (Figure 5). There were no postoperative stroke events or restenosis in this subgroup.

## DISCUSSION

Carotid revascularization has become an important treatment option for patients with arteriosclerotic carotid disease. Initially, the superiority of carotid surgery over medical treatment was demonstrated,^11^ but carotid stenting later came to be considered a safe and effective alternative.^1^^,^^2^

Taking into account population aging, with the increase in comorbidities that this entails, a transition from CEA to CAS is currently taking place, especially in high-risk patients precluded from CEA and symptomatic patients who were not previously offered surgical treatment and for whom CAS could now be an option.

Sex plays an important role in cardiovascular diseases. Men have a higher prevalence and incidence of strokes, but strokes in women tend to be more severe.^14^ This raises the question of whether the postoperative behavior of CAS might differ according to sex.

Initially, female sex was associated with a higher stroke rate after CEA.^3^ Many of the clinical trials focused on open surgery were limited by the low proportion of women included and the long-term benefit was affected by perioperative comorbidity in women.

The SAPPHIRE^1^ trial was the first to demonstrate the non-inferiority of CAS versus CEA, but did not compare results between sexes. Years later, higher rates of restenosis, stroke or re-intervention were reported in women after CEA and CAS.^4^^,^^15^^,^^16^ However, there were also authors who did not confirm these findings.^7^^,^^17^^-^19 Bisdas et al.^14^ reported evidence of higher rates of mortality and adverse events (myocardial infarction and stroke) in symptomatic women after CAS. However, they showed comparable results between asymptomatic men and women.

Despite the fact that women have been considered a subgroup of patients where the benefit of carotid surgery is more questionable (the benefit is obtained at 10 years, and not at 5 years as happens in men),^12^ the results of our series do not confirm these findings and we do not observe differences between groups: our outcomes are similar in the male and female sex.

Our study specifically analyzed the findings of our center after CAS according to sex. In our cohort, demographic variables were comparable between the sexes.

Almost half of our cases (29 patients) were treated during the acute phase of the disease (on a stroke code) and the rest of the cases either had a history of hostile neck (previous surgery or radiotherapy), or had severe comorbidities. Postoperative adverse events in women were comparable with those observed in men and there were no significant differences, with similar rates of stroke, myocardial infarction, and death.

On the other hand, restenosis due to neointimal hyperplasia has been described during the first 12-18 months after the procedure and our series did not demonstrate statistically significant differences between the sexes during this period. In fact, there were 5 patients with restenosis: 4 men *vs.* 1 woman, p=0.9693; and only 2 of them required reintervention.

It should be noted that four of the 10 patient deaths that occurred during follow- were patients with acute symptoms who died from the cerebrovascular event for which they were admitted. Another patient died due to a known neoplasm. Two patients died of cerebrovascular causes during follow-up and three patients died from other non-cardiovascular causes.

Several recent studies also showed that there is no greater risk of complications associated with female sex. Mayor et al.^7^ found no significant differences in adverse events in asymptomatic women, although they did report an increased risk of stroke in symptomatic women (including CEA and CAS). Jim et al.^17^ analyzed a sample of 9865 patients and did not observe an increased risk of 30-day events in women, while Goldstein et al.^18^ also showed no differences in cardiological or neurological events or death at 30 days or 5 years. Casana et al.^19^ did not report significant differences between men and women in long-term adverse events.

It is possible that some differences in the literature could be due to the diversity of follow-up criteria (30-60 days after the procedure or at 1-5 years) or to improvement of the carotid revascularization technique and the best medical treatment.

There are several limitations to our study. Primarily, the small sample size; we acknowledge that this is an observational non-randomized single-center study that covered 3.5 years. The retrospective nature of the study may have conditioned data collection and since it was not a randomized study, stent selection was at the surgeon’s discretion. Also, both urgent (stroke code) and scheduled patients were included, although statistical analysis was performed for both subgroups separately and no significant differences were found in either subgroup. The subsets were grouped to avoid further reducing subgroup sample sizes.

In conclusion, despite the limitations of our study, CAS is a therapeutic option that is as effective and safe in women as in men, with similar results between them in our sample. No sex differences were observed.
